# *Paenibacillus larvae* and their phages; a community science approach to discovery and initial testing of prophylactic phage cocktails against American Foulbrood in New Zealand

**DOI:** 10.20517/mrr.2023.16

**Published:** 2023-08-01

**Authors:** Danielle N. Kok, Diana Zhou, Philippos K. Tsourkas, Heather L. Hendrickson

**Affiliations:** ^1^School of Natural Sciences, Massey University, Auckland 0632, New Zealand.; ^2^School of Biological Sciences, University of Canterbury, Christchurch 8140, New Zealand.; ^3^Department of Biostatistics and Medical Informatics, School of Medicine and Public Health, University of Wisconsin, Madison, WI 53792, USA.

**Keywords:** *Apis mellifera*, phage, environmental sampling, community science

## Abstract

**Background:** American foulbrood (AFB) is a devastating disease of the European honey bee (*Apis mellifera*) and is found throughout the world. AFB is caused by the bacterium *Paenibacillus larvae* (*P. larvae*)*.* Treatment with antibiotics is strictly forbidden in many regions, including New Zealand. Safe and natural prophylactic solutions to protect honey bees from AFB are needed. Bacteriophages are a well-studied alternative to antibiotics and have been shown to be effective against *P. larvae* in other countries.

**Methods:** We employed a community science approach to obtaining samples from around New Zealand to discover novel bacteriophages. Standard isolation approaches were employed for both bacteria and bacteriophages. Host range testing was performed by agar overlay spot tests, and cocktail formulation and *in vitro* testing were performed in 96-well plate assays, followed by sub-sampling and CFU visualization on agar plates.

**Results:** Herein, we describe the discovery and isolation of eight *P. larvae* bacterial isolates and 26 *P. larvae* bacteriophages that are novel and native to New Zealand. The phage genomes were sequenced and annotated, and their genomes were compared to extant sequenced *P. larvae* phage genomes. We test the host ranges of the bacteriophages and formulate cocktails to undertake *in vitro* testing on a set of representative bacterial strains. These results form the basis of a promising solution for protecting honey bees in New Zealand from AFB.

## INTRODUCTION

The European honey bee (*Apis mellifera*) is a valuable livestock animal globally^[1]^. In New Zealand, this value comes from their role in the pollination of horticultural and agricultural crops_,_ which contributes roughly 8.7 billion dollars to New Zealand’s current GDP per annum, assuming this ratio has maintained since 2013^[2]^. The export of apiculture products, including honey, beeswax and live bees, contributes a further $483 million NZD p.a.^[3]^. Since 2006, New Zealand has seen a steep increase in the number of beekeepers and apiaries; with these rising numbers, there has also been a rising number of colony losses observed^[3]^.

Honey bees are under constant attack by abiotic and biotic factors, including but not limited to herbicides, pesticides, parasites, viruses and bacteria^[4]^. The two biggest biotic threats to honey bees today are the parasitic Varroa mite (*Varroa destructor*) and American foulbrood (AFB), which is caused by the spore-forming, bacterial pathogen *Paenibacillus larvae* (*P. larvae*). AFB is a serious and destructive disease that attacks honey bees in their larval and pupal stages^[5,6]^. AFB has detrimental consequences at both the larval and colony level^[7-9]^.

AFB has been present in New Zealand for at least 146 years after first being discovered in 1877^[10,11]^. By 1887, AFB had caused significant damage around the country and led to a 70% reduction in honey production^[12]^. The use of antibiotics to treat or mask an AFB infection in New Zealand is strictly prohibited^[13,14]^. Current legislation stipulates that beekeepers must destroy hives infected with AFB within seven days of discovery, using petrol fumes and incineration to ensure all traces of AFB are removed^[13,14]^. This method is costly to both the beekeeping community and the New Zealand economy.

A potential solution to AFB infection in New Zealand is the prophylactic application of bacteriophages in a phage cocktail. Bacteriophages, or phages informally, are self-propagating viruses that are only able to infect and replicate within bacteria. Phages are ubiquitous and are the most numerous biological entity on Earth, with at least 10^31^ phages in existence globally at any point in time^[15,16]^.

Work undertaken overseas has shown it is possible to protect honey bee larvae from AFB infection through the application of *P. larvae* phage cocktails both *in vitro*^[17]^ and in honeybee colonies in an at-risk apiary^[18]^. In the latter, phage protection appeared to remain intact for at least four months after application of the cocktail. In addition, a recent genomic analysis was performed in which 48 completely sequenced *P. larvae* phages were described and classified into four clusters and one “singleton”^[19]^. These provide a rich resource of information on phage diversity. However, New Zealand biosecurity laws prohibit us from importing non-native phages for domestic release. Therefore, in order to create phage cocktails to protect honeybees in our domestic honey production sector, it is necessary that we isolate *P. larvae* phages that are native to New Zealand. While a collection of *P. larvae* bacterial isolates was previously reported^[20]^, that collection was subsequently destroyed (*P. Lester personal communication*). Discovering novel New-Zealand-based *P. larvae* isolates was therefore necessary.

Honeybees are primarily kept in New Zealand for the production of high-value honey, like mānuka (*Leptospermum scoparium*) and rātā (*Metrosideros robusta*), not for their pollination services. As a result, honeybee colonies are often located in rough terrain or on private property^[11]^. New Zealand is also roughly the size of Great Britain or Japan, so sampling from across this geographic space was a technical challenge. Accessing honeybee colonies and their detritus to search for phages would have been extremely onerous for our science team. We therefore chose to use a community science approach to phage discovery. This approach simplified our sampling regime, but more importantly, it allowed us to begin to communicate the value of our project directly to the beekeepers through an infographic, face-to-face interactions, and speaking at local and national stakeholder meetings.

Herein, we describe how we developed a collection of novel *P. larvae* bacterial isolates and used these to discover *P. larvae* phages native to New Zealand. We briefly describe their genome sequences, report phage host ranges, and design phage cocktails in addition to performing *in vitro* testing of several phage cocktails. This work forms the groundwork to develop an approach to protecting beehives using New Zealand native phages that can be applied to protect hives against infection by a devastating bacterial pathogen that is affecting this industry globally.

## MATERIALS AND METHODS

### Isolation of *Paenibacillus larvae*

*P. larvae* was isolated by swabbing suspected brood frames and wiping swabs on MYPGP^[21]^ with Nalidixic acid (10 μg/mL) and Pipemidic acid (10 μg/mL) plates. Plates were incubated at 37 ^o^C for 3-5 days until colonies had formed. Single colonies were picked and purified by single colony isolation on another MYPGP plate. Subsequently single colonies were picked and grown in liquid MYPGP for 48 h at 37 ^o^C and shaken at 100 rpm, then frozen at -80 ^o^C.

### Sporulation of *Paenibacillus larvae* for microscopy

To produce bacterial spores for microscopy, a 10-fold dilution series of *P. larvae* PFR-Pl-2006 bacterial culture was spread onto several MYPGP agar plates. Plates were incubated at 37 ^o^C for 6-7 days and plates exhibiting individual colonies were selected. After incubation, spores were removed from the plates by washing with 5 mL cold sterile water. Water was added to the plate, and the surface of the plate was gently scraped with a sterile inoculation loop to loosen spores. Water and spores were then removed from the plates using a syringe and transferred into Eppendorf tubes. The spore suspension was concentrated via centrifugation (12,000 × g, 15 min, 4 ^o^C). After centrifugation, the supernatant was discarded, and the spore pellet was resuspended in 1 mL of cold water. This step was repeated three times. The final spore pellets from all tubes were resuspended in a total volume of 2 mL cold water. Spores were stored at 4 ^o^C^[22]^.

### Bacterial DNA extraction and 16S rRNA PCR

Bacterial DNA was extracted from overnight cultures in mBHI (Oxoid CM1135B) broth using the commercially available Promega Wizard Genomic DNA Purification kit (www.promega.com/protocols/). The protocol for gram-positive bacteria was followed. A PCR mix was prepared with each tube containing a final volume of 50 μL. The amplification conditions were 95 ^o^C (3 min) followed by 30 cycles of 93 ^o^C (1 min), 55 ^o^C (30 s), and 72 ^o^C (1 min); and a final cycle of 72 ^o^C for 5 min. PCR products were visualized on a 1% agarose gels run at 120 volts for 30 min.

Primers used were^[23]^:

AFB-F 5’-CTT-GTG-TTT-CTT-TCG-GGA-GAC-GCC-A-3’

AFB-R 5’-TCT-TAG-AGT-GCC-CAC-CTC-TGC-G-3’

### Bacterial DNA sequencing and assembly

DNA was either sent for sequencing at MicrobesNG (Birmingham, UK) or MiGS (Pittsburgh, PA, USA) for complete genome Illumina sequencing. Sequencing was performed by MicrobesNG by preparing genomic DNA libraries with Nextera XT Library Prep Kit (Illumina, San Diego, USA). Libraries were then sequenced on an lllumina NovaSeq 6000 (Illumina, San Diego, USA) using a 250 bp paired-end protocol (www.microbesng.com). Sequencing was performed by MiGS on the NextSeq 2000 platform (Illumina Inc., San Diego, CA, USA) using a paired-end library (www.seqcenter.com). Genomes were assembled using SPAdes 3.15.3^[24,25]^ and then annotated using either RAST 1.073^[26-28]^ or Prokka 1.14.5^[29]^. Average coverage was 30x with 157-219 contigs assembled.

### Processing soil/hive samples for phages

Soil samples were processed as previously described^[17]^. Only one pass through a 0.45 μm sterile syringe filter was performed. The resulting filtrate was used as a starting material for enrichment. Enrichments were a combination of 1 mL of starting material, 100 μL of each of eight *P. larvae* bacterial isolates, 8 mL mBHI and 0.4% glucose. These were incubated for 48 h at 37 ^o^C, shaken at 100 rpm. After 48 h, enrichments were centrifuged at 3,200 g for 15 min, and filter sterilized to 0.45 μm. The resulting supernatants were assayed for phage presence by 3 μL spots on double-layer agar containing one of the *P. larvae* bacterial isolates.

### Phage plaque purification

Phages underwent three rounds of purification. Plaques were picked off a double-agar plate using a 200 μL pipette tip; the tip was put in 100 μL of BHI and pipetted up and down to remove phage particles. This lysate was used to inoculate the next double-agar plate.

### Creation of lysates

To create phage lysates, 10 plaque plates with the highest number of individual plaques were flooded with 8 mL of BHI. Plates were left to sit at room temperature for 2 h. At the end of 2 h, plates were swirled, and the lysate was removed. Lysates were filtered with a 0.45 μm filter and pooled in a 50 mL falcon tube. Titers were increased using the Rapid Adaptive Mutation of Phage RAMP-UP protocol^[30]^.

### Phage DNA extraction and sequencing

Phage DNA was extracted using a modified zinc chloride precipitation method^[31]^. Modifications included the addition of 1 µL Proteinase K (20 mg/mL), incubated at 37 °C for 10 min after the resuspension in TES buffer (0.1M Tris-HCl, pH 8, 0.1M EDTA, 0.3% SDS) step. Tubes were left overnight on ice after isopropanol was added. 1 µL of pure glycogen was added to each tube at the beginning of Day 2 before the centrifugation step to aid in pelleting of DNA. DNA pellets were resuspended in 50 µL nuclease-free water.

Phage genomes were sequenced and annotated as previously described^[30]^. Briefly, phage genomes were assembled using Geneious 9.05 (Auckland, New Zealand) (https://www.geneious.com); assembled genomes were then run through Phage Commander^[32]^ to identify all genes. Genomes were manually checked using DNA Master^[33]^ and as previously described^[34]^.

### Host range testing

The ability of phages to infect each isolate was assessed by 3 μL spots of each phage lysate on double-layer agar containing 500 μL of bacterial lawn. Each *P. larvae* bacterial isolate was tested separately. The majority of spot tests showed the presence of individual plaques owing to low phage titers during this testing.

### *In vitro* cocktail assays

Phage titers were normalized to 1 × 10^8^ PFU/mL and 50 μL of each phage selected was combined into a cocktail. Bacterial cultures were grown in BHI for 48 h at 37 ^o^C to ~1 × 10^8^ CFU/mL. The bacteria were serially diluted up to a 10^-6^ dilution and 20 μL was aliquoted into 96-well plates containing 90 μL 2 × BHI and 90 μL BHI. The phage cocktails were also serially diluted such that each row contained from 10^7^ to 10^3^ PFU total phage. 20 μL of the phage cocktail was added to each well of the plate. Plates were incubated, shaking at 37 ^o^C for 24 h. Aliquots of 3 μL were spotted onto BHI plates and incubated for three to four days at 37 ^o^C to observe CFU.

## RESULTS

### Isolating *P. larvae* from infected colony material

**Figure 1:**
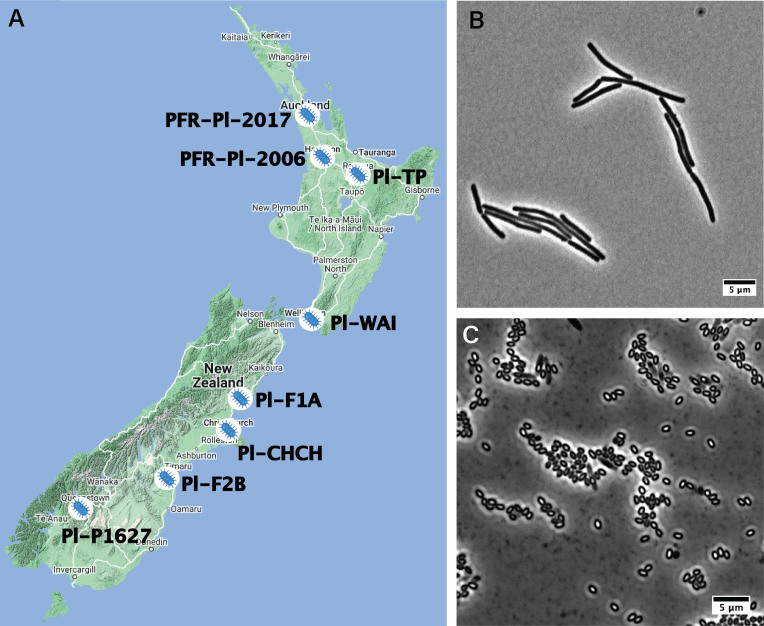
*Paenibacillus larvae* bacterial strains (A) Locations of the eight isolated *Paenibacillus larvae* (*P. larvae*) bacterial strains; (B) Vegetative form of *P. larvae* PFR-Pl-2006; (C) Spore form of *P. larvae* PFR-Pl-2006*.* Scale bar: 5 μm.

Previous work suggested that a curated collection of *P. larvae* isolates from New Zealand had been characterized^[20]^. Further investigation revealed that the existing collection had been destroyed (*P. Lester private communication*). Therefore, a new collection of representative *P. larvae* strains was needed. AsureQuality, a New Zealand government-approved testing facility, provided us with swabs of brood frames or infected larvae material and whole brood frames from beehives suspected of AFB infection. Potential isolates were cultured on semi-selective MYPGP agar plates in order to obtain single colonies. Ultimately, eight *P. larvae* strains were isolated from around New Zealand, with each isolate coming from a different location [Figure 1A and Table 1]. *P. larvae* is a filamentous (2.5-5 μm by 0.5-0.8 μm), spore-forming, gram-positive bacterium [Figure 1B and C]^[6]^. Isolates were confirmed to be *P. larvae* by positive amplification with 16S rRNA PCR primers^[23]^.

**Table 1:** *Paenibacillus larvae* bacterial strains isolated from New Zealand

***P. larvae* strain**	**Isolation location**	**MLST ST**	**GC%**	**No. of** >**contigs**	**Size range contigs** **(Kbp)**	**Accession No.**
**Pl-WAI**	Wellington	18	44.2%	219	0.128-218	JARDRH000000000
**Pl-TP**	Rotorua	18	44.1%	157	0.128-250	JARDRJ000000000
**Pl-CHCH**	Christchurch	18	44.1%	163	0.128-218	JARDRI000000000
**PFR-Pl-2017**	Auckland	18	44.1%	176	0.128-218	JARDRG000000000
**PFR-Pl-2006**	Hamilton	18	44.1%	185	0.128-218	JARDAI000000000
**Pl-F1A**	North Canterbury	18	44.1%	175	0.5-191	JARDRL000000000
**Pl-F2B**	South Canterbury	18	44.0%	167	0.5-191	JARDRM000000000
**Pl-P1627**	Queenstown	23	44.1%	171	0.5-195	JARDRK000000000

*P. Larvae: Paenibacillus larvae.*

DNA was extracted from each of the bacterial isolates and submitted for genome sequencing. Genomes were assembled using SPAdes 3.15.3^[24,25]^ and then annotated using either RAST 1.073 ^[26-28]^ or Prokka 1.14.5^[29]^. The resulting genome assemblies had a range of 157 to 219 contigs, with sizes varying from 0.12 to 218 Kbp. These *P. larvae* strains had GC contents of 44.0% to 44.2% [Table 1].

Multilocus sequence typing (MLST) was undertaken using PubMLST^[35]^. MLST for *P. larvae* consists of the following seven housekeeping genes: *ftsA* (cell division protein), *clpC* (catabolite control protein A), *glpT* (glycerol-3-phosphate permease), *glpF* (glycerol uptake facilitator protein), *rpoB* (RNA polymerase beta subunit), *Natrans* (forward sodium dependant transporter), and *sigF* (sporulation sigma factor F) as these offered the most diversity between genomes tested^[36]^. Seven of the New Zealand isolates belonged to the 18 MLST ST and one belonged to 23 MLST ST [Table 1]. MLST can also be used to distinguish between the ERIC I genotype and the ERIC II genotype. MLST 18 and MLST 23 both belong to the ERIC I genotype^[36-38]^.

We used CRISPRFinder to look for detectable CRISPR systems in these eight isolates^[39]^. Seven of the isolates contained four CRISPR arrays and one isolate contained five. The total number of spacers within the CRISPR arrays for each isolate varied from 15-25 spacers [Table 2]. Across all eight isolates, 29 unique spacers were observed, Pl-P1627 contained 12 unique spacers that were not found in any of the other isolates.

**Table 2:** CRISPR array and spacer details of the eight *Paenibacillus larvae* isolates

***P. larvae* strain**	**No. of CRISPR arrays**	**No. of spacers**	**No. of unique spacers**
**Pl-WAI**	4	15	0
**Pl-TP**	4	17	0
**Pl-CHCH**	4	17	0
**PFR-Pl-2017**	4	17	0
**PFR-Pl-2006**	4	16	0
**Pl-F1A**	4	17	0
**Pl-F2B**	4	17	0
**Pl-P1627**	5	25	12

*P. Larvae: Paenibacillus larvae.*

We also used DefenseFinder^[40,41]^ to search for known anti-phage systems in our bacterial strains. All eight isolates contained the same seven anti-phage systems: both a type I and II restriction-modification system^[42]^, a Gao_let system^[43]^, two Cas systems (CAS_Class1-Subtype-III-B and CAS_Class1-Subtype-I-B)^[44]^, a Wadjet_III system^[45]^, and a Mokosh_TypeII system^[46]^.

Finally, we used Phaster^[47,48]^ to identify prophages contained within the genomes. Phaster designates prophages as either intact, questionable or incomplete by comparing them to a NCBI database of complete viral genomes. Potential prophage regions are then given a completeness score; this score is calculated on the proportion of phage genes in the identified region. An intact prophage has a score > 90, a questionable prophage has a score between 70-90, and an incomplete prophage has a score < 70. All isolates contained at least one intact prophage, with six containing two intact prophages. The intact prophages were genomically similar to *P. larvae* Phage Harrison and Phage Vegas^[49]^. All genomes also contained 3-4 questionable prophages and 6-9 incomplete prophages [Table 3].

**Table 3:** Prophages found in the eight *Paenibacillus larvae* isolates

***P. larvae* strain**	**Total prophages**	**Intact**	**Name of intact phage**	**Size of prophage (Kb)**	**Total proteins #**	**GC content (%)**	**Questionable**	**Incomplete**
**Pl-WAI**	14	2	Vegas	41.7	60	43.06	3	9
			Harrison	15.2	19	43.30		
**Pl-TP**	14	2	Vegas	39.6	61	43.29	3	9
			Harrison	15.2	18	43.29		
**Pl-CHCH**	14	2	Vegas	39.6	61	43.30	3	9
			Harrison	15.2	18	43.31		
**PFR-Pl-2017**	13	2	Vegas	39.6	60	43.29	4	7
			Harrison	15.2	18	43.30		
**PFR-Pl-2006**	15	2	Vegas	39.6	61	43.30	4	9
			Harrison	15.2	18	43.30		
**Pl-F1A**	12	2	Vegas	35.7	55	43.56	3	7
			Harrison	31.5	52	42.07		
**Pl-F2B**	13	1	Harrison	41.5	81	41.95	3	9
**Pl-P1627**	10	1	Vegas	43.1	66	43.68	3	6

*P. Larvae: Paenibacillus larvae.*

### Phage discovery

#### A community science approach to national sample collection

**Figure 2:**
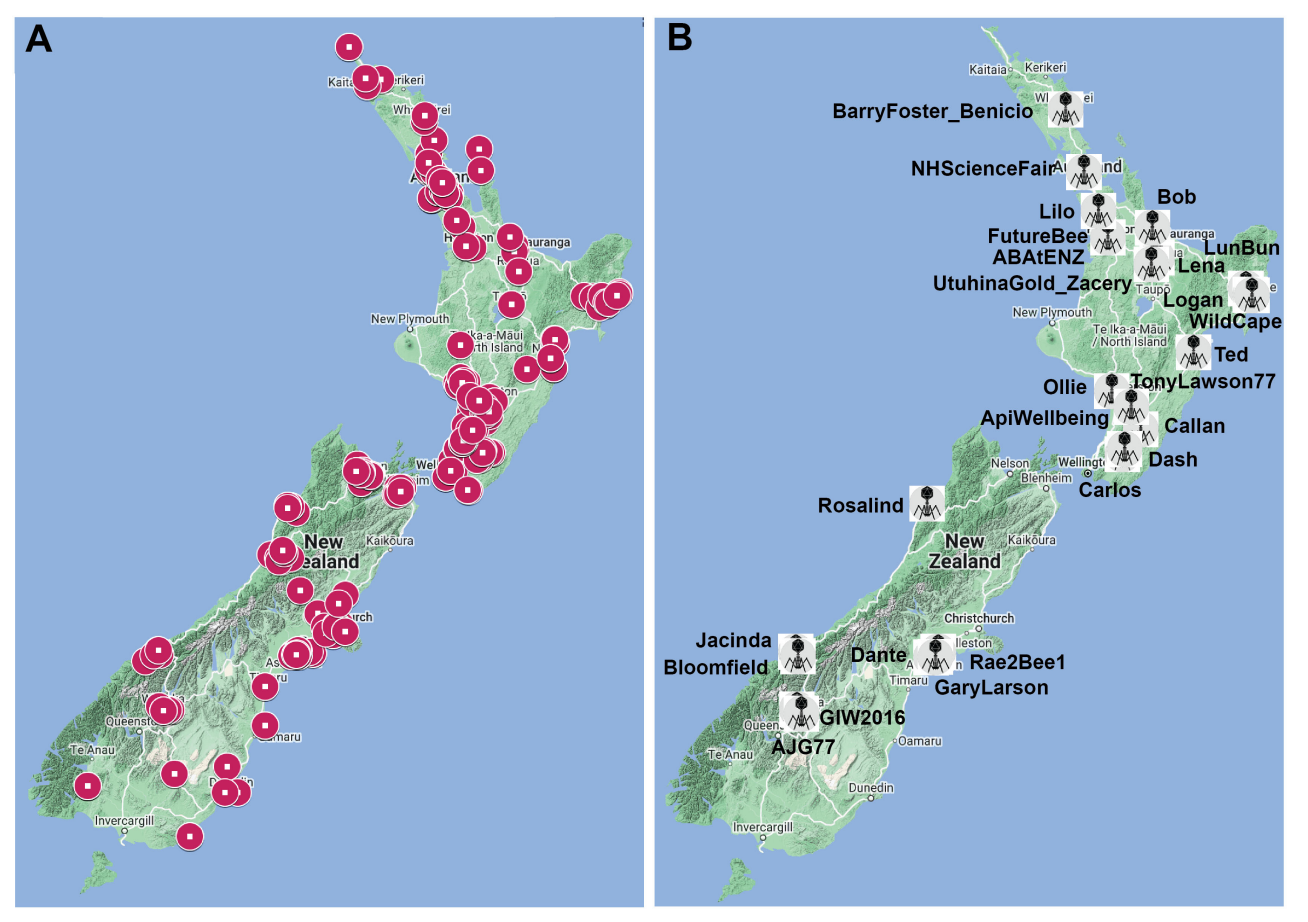
Sourcing samples from beehives across the nation. (A) Locations of samples of soil, bee debris, or wax that were provided by beekeepers; (B) The locations and names of *Paenibacillus larvae* (*P. larvae*) phages discovered as a result of these efforts.

Bee hives are distributed throughout the country in out-of-the-way locations and often on private property. In order to isolate phages from around New Zealand, we used a community science approach to engage the assistance of New Zealand beekeepers. An infographic [Supplementary Figure 1] was developed and distributed widely in beekeeping circles via social media, beekeeping magazines, in-person apiculture conferences and posted on our website (http://www.hendricksonlab.co.nz/ABATE/). Beekeepers were encouraged to take samples of soil or hive/bee debris and return them to be processed for the presence of phages in a prepaid and addressed envelope. As part of the community science, beekeepers were able to name any phages that were discovered within a sample they had provided us. A total of 720 sample tubes were distributed, out of which 430 samples were returned and processed, with a return rate of 60%. Samples were taken from a wide distribution of locations in New Zealand [Figure 2A].

Twenty-six of the samples contained a novel phage able to infect at least one of our bacterial isolates of *P. larvae* [Figure 2B and Table 4]. These phages were generally of a low titer (10^2^-10^7^ mL^-1^), which initially prevented us from progressing to genome sequencing and electron microscopy as our typical methods require 10^9^ mL^-1^. We therefore developed the Rapid Adaptive Mutation of Phage-UP or RAMP-UP protocol to increase phage titer by mutation, which can work in as little as four days^[30]^.

**Table 4:** Details of 26 *Paenibacillus larvae* phages discovered, sequenced and annotated

	**Geographic region**	**Bacteria isolated on**	**Genome length (bp)**	**No. of genes**	**GC content (%)**	**Cluster**	**Accession No.**
**ABAtENZ**	Hamilton	Pl-PFR-2017	44,419	82	42.97	Vegas	OP503968
**AJG77**	Wanaka	Pl-PFR-2017	44,417	82	42.98	Vegas	OP503969
**ApiWellbeing**	Masterton	Pl-F1A	44,429	82	43.01	Vegas	OP503970
**BarryFoster_Benicio**	Whangarei	Pl-F1A	44,421	82	42.98	Vegas	OP503543
**Bloomfield**	Haast	Pl-PFR-2017	44,419	82	42.98	Vegas	OP503971
**Bob**	Matakana Island	F2B	43,553	80	43.03	Vegas	OP503972
**Callan**	West Taratahi	Pl-PFR-2006	44,768	77	39.69	Harrison	OP503989
**Carlos**	Carterton	Pl-F1A	44,430	83	42.98	Vegas	OP503973
**Dante**	Elgin	Pl-WAI	44,420	82	42.98	Vegas	OP503974
**Dash**	West Taratahi	Pl-PFR-2006	44,599	79	39.39	Harrison	OP503990
**FutureBee**	Hamilton	Pl-TP	44,417	83	42.98	Vegas	OP503975
**GaryLarson**	Willowby	Pl-F2B	44,420	82	42.98	Vegas	OP503976
**GIW2016**	Wanaka	Pl-PFR-2017	43,555	80	43.01	Vegas	OP503977
**Jacinda**	Haast	Pl-PFR-2017	44,419	82	42.97	Vegas	OP503978
**Lena**	Rotorua	Pl-PFR-2017	44,420	82	42.97	Vegas	OP503979
**Lilo**	Pukekawa	Pl-F1A	40,941	70	40.33	Harrison	OP503991
**Logan**	Tolaga Bay	Pl-PFR-2017	44,419	82	42.99	Vegas	OP503980
**LunBun**	Gisborne	Pl-F1A	44,421	82	42.97	Vegas	OP494865
**NHScienceFair**	Albany	F1A	44,419	82	42.98	Vegas	OP503981
**Ollie**	Marton	Pl-PFR-2017	44,420	83	42.98	Vegas	OP503982
**Rae.2Bee1**	Fairton	Pl-TP	44,420	82	42.97	Vegas	OP503983
**Rosalind**	Westport	F1A	43,556	80	43.00	Vegas	OP503984
**Ted**	Napier	Pl-PFR-2017	44,419	82	42.99	Vegas	OP503985
**TonyLawson77**	Palmerston North	Pl-F1A	44,420	82	42.96	Vegas	OP503986
**UtuhinaGold_Zacery**	Rotorua	Pl-PFR-2017	44,420	82	42.97	Vegas	OP503987
**WildCape**	Gisborne	Pl-F1A	44,430	82	43.00	Vegas	OP503988

*P. Larvae: Paenibacillus larvae.*

**Figure 3:**
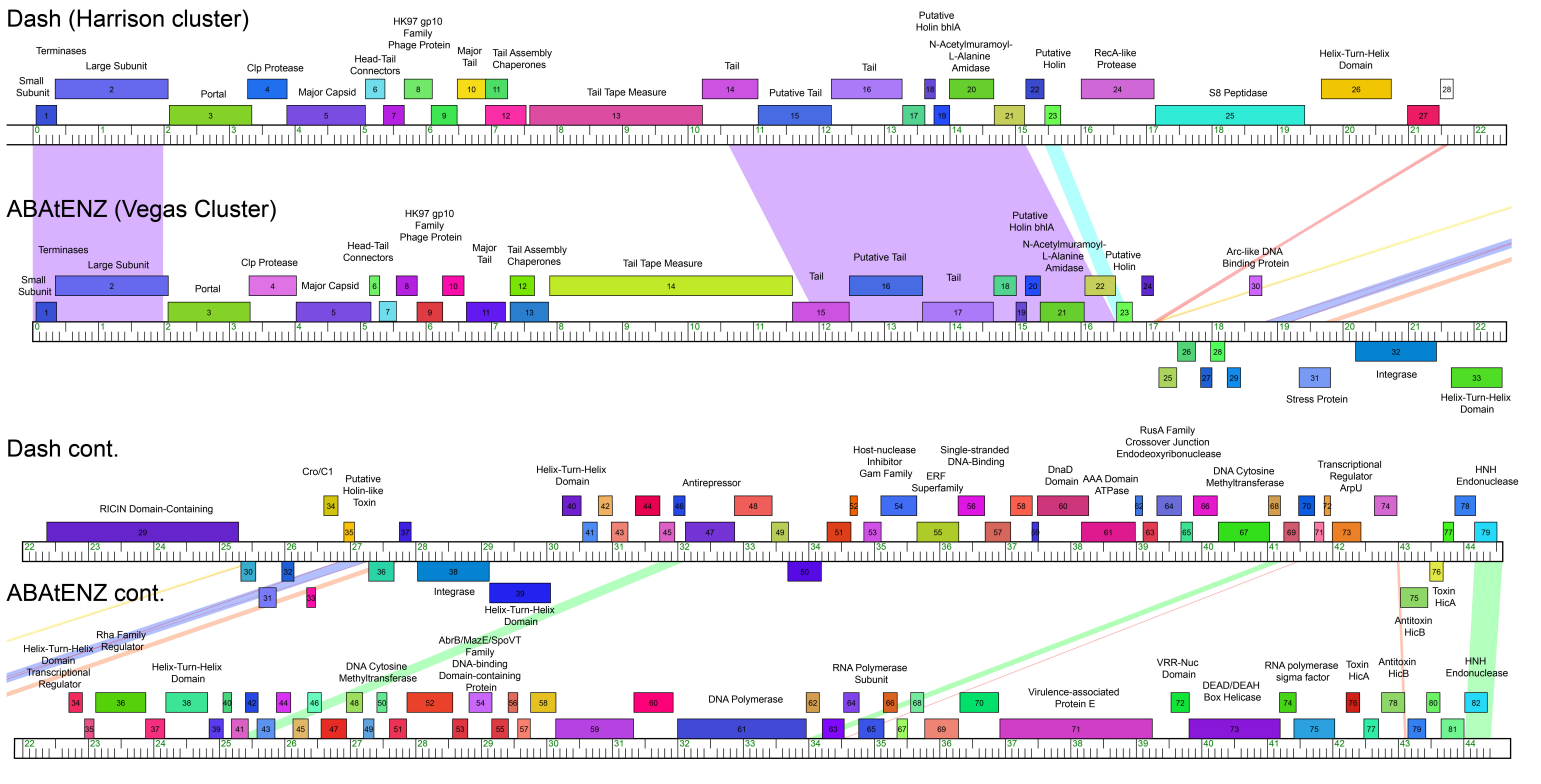
Representative phage genome maps for Dash (Harrison cluster) and ABAtENZ (Vegas cluster) generated with Phamerator.org, showing pairwise sequence similarity (shading) and the homologous genes (matching-colored boxes). The shades of color indicate a combination of length and significance of nucleotide identity, and the large purple blocks are the strongest regions of similarity observed between phages Dash and ABAtENZ.

Upon sequencing, we discovered the New Zealand phages were between 40-44 kbp in length with 70-83 genes per genome. The phages belong to two of the four major genomically determined clusters of *P. larvae* phages; three belong to the Harrison cluster and 23 belong to the Vegas cluster [Table 4]*.* Clusters were determined by average nucleotide identity (ANI); if two phages have ANI greater than or equal to 60%, they are placed in the same cluster^[19]^. All New Zealand *P. larvae* phages are linear and use the 3’ cohesive end DNA packaging mechanism, similar to the majority of previously described *P. larvae* phages^[19]^. The New Zealand *P. larvae* phages are lytic *in vitro,* despite the presence of annotated integrases in their genomes, the presence of which suggests the capacity for a temperate lifestyle. Phage Dash (Harrison Cluster) has an integrase at GP38 and phage ABAtENZ (Vegas Cluster) has an integrase at GP32 [Figure 3]. The presence of integrases, and the absence of evidence of a temperate lifecycle in the laboratory, is consistent across the majority of known *P. larvae* phages^[50]^. All New Zealand phage genomes encode a conserved N-acetylmuramoyl-L-alanine amidase that enables them to lyse their host, similar to all other *P. larvae* phages^[19]^. Phages Dash and Lilo encode a large (975 amino acids) Plx1 toxin that confers virulence to their host^[51,52]^. The presence of this toxin in the genomes of these two phages automatically makes them unsuitable for therapy applications.

#### Isolating phages from soil/hive material

**Figure 4:**
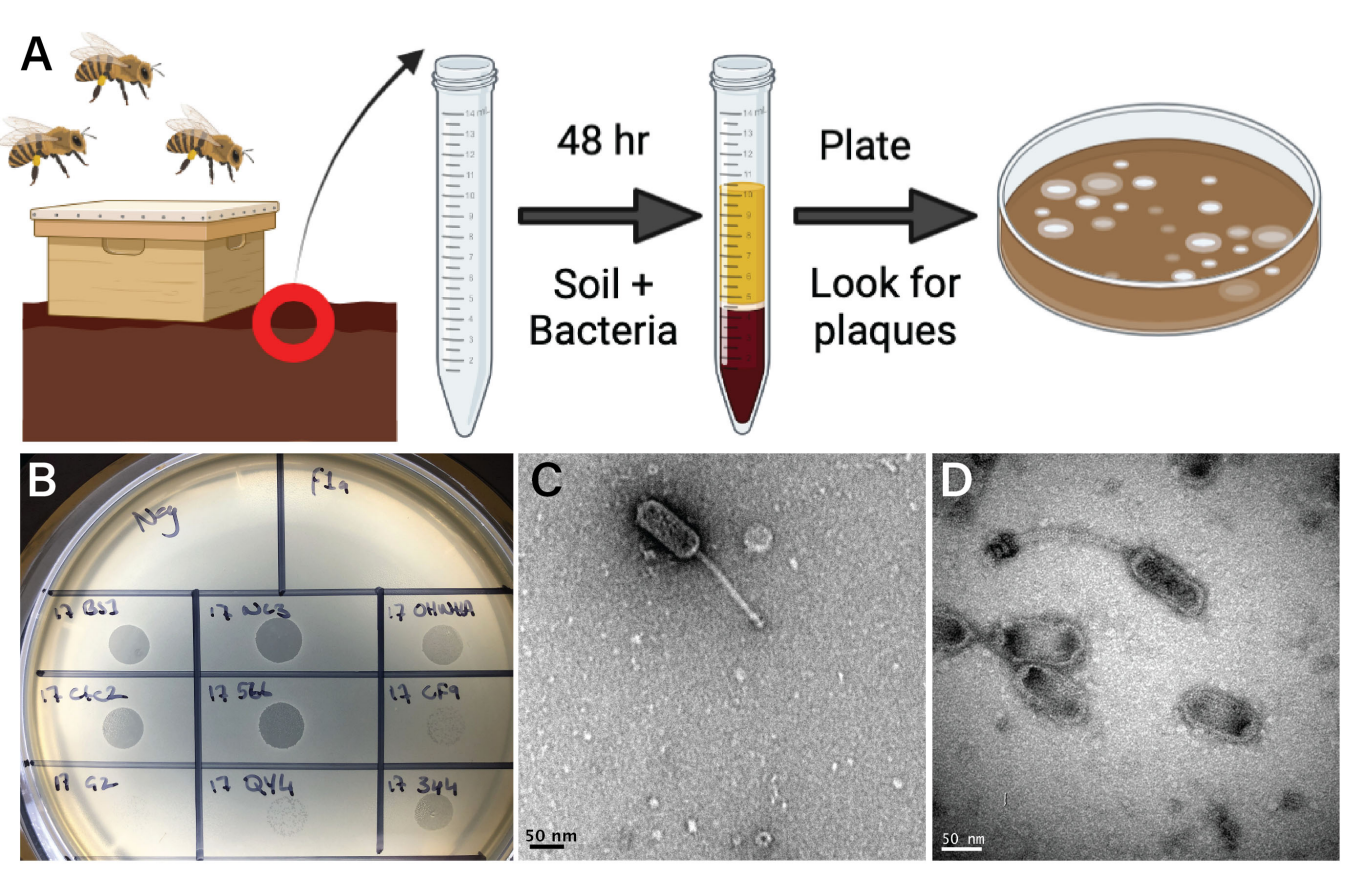
Phage discovery (A) Schematic of phage enrichment and isolation process (created with BioRender.com); (B) Positive spot tests after enrichment; (C) Representative TEM image of Phage Lilo (Harrison cluster); (D) Representative TEM image of Phage Ollie (Vegas cluster). Scale bars = 50 nm. TEM: Transmission electron microscopy.

The 430 samples received were processed using an enrichment technique followed by three rounds of plaque purification [Figure 4A and B]. The plaque morphologies of all isolated phages were tiny, pin-prick plaques that appeared clear. When tested by a standard spot titer plate method, all of our phages had very low effective titers ranging from 6.7 × 10^2^ to 2.7 × 10^5^. A RAMP-UP technique was used to increase the titer of all phages in order to extract DNA and send it for sequencing^[30]^. Once these titers were raised by this method to > 1 × 10^8^, we proceeded with visualization and complete genome sequencing.

#### Transmission electron microscopy of isolated phage

Electron microscopy was undertaken on Phage Lilo (Harrison Cluster) Figure 4C and Phage Ollie (Vegas Cluster) [Figure 4D]. These two phages were selected as they each represented one of the two clusters of phages found in New Zealand. These revealed phages with long, filamentous, non-contractile tails; phages with these types of tails are classified as having Siphoviridae morphology^[53]^. All the new phages reported here are *Gochnauervirinae,* a subfamily in the *Caudoviricetes* class. All but one of the known *P. larvae* phages have this morphotype^[19]^. Phage Lilo had a tail of approximately 148 nm in length, with a prolate head measuring approximately 105 nm by 41 nm. Phage Ollie had a tail approximately 156 nm in length, with a prolate head measuring approximately 106 nm by 43 nm.

### Host range testing

**Figure 5:**
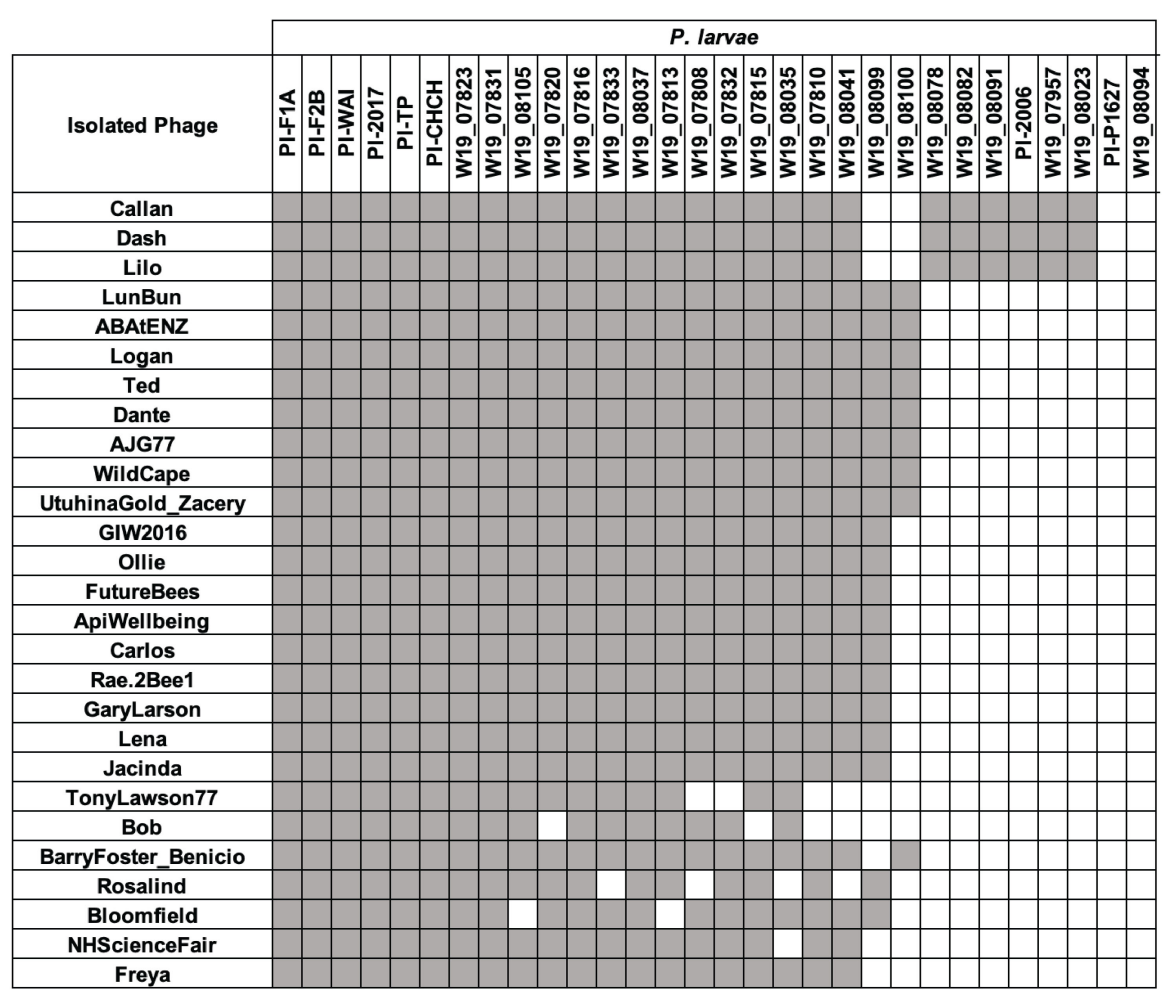
Host range of *Paenibacillus larvae* (*P. larvae*) phages on *P. larvae* bacterial isolates from New Zealand. Grey boxes indicate cell lysis and white boxes indicate no cell lysis has occurred. NB: In some instances, spot clearing was observed, but plaques were not. This is explicit in Supplementary Figure 2.

Specificity of the New Zealand isolated phages on each of the eight native *P. larvae* isolates identified in this paper, as well as 22 native *P. larvae* isolates provided by the ApiWellbeing team^[54]^ was carried out using standard spot test assays. Phages were scored as positive or negative for cell lysis. Nine distinct infection patterns were identified [Figure 5].

None of the phages were capable of lysing all 30 bacterial isolates, but they were able to lyse between 57% to 87%. Bacterial isolatets Pl-P1627 and W19_08094, both isolated from the Otago region, are not lysed by any of the phages found in New Zealand to date. Pl-P1627 belongs to a different multilocus sequence type than the other seven bacterial strains identified and sequenced in this paper, as well as having 12 unique spacer sequences within its CRISPR arrays.

Phages Dash, Lilo, and Callan can infect *P. larvae* strains W19_08078, W19_08082, W19_08091, PFR-Pl-2006, W19_07957, and W19_08023 which are not lysed by any other phage. W19_08099 and W19_08100 are not able to be infected by these three phages, which otherwise infect all non-resistant bacteria. *P. larvae* isolates Pl-F1A, Pl-F2B, Pl-WAI, Pl-2017, Pl-TP, Pl-CHCH, W19_07823, and W19_07831 are lysed by all phages in this study. *P. larvae* isolate W19_08100 is lysed by nine of the phages. Phages TonyLawson77, Bob, and Rosalind have the smallest ranges of infectivity and are only capable of lysing 57% of the strains.

### Cocktail formulation and *in vitro* testing

Initially, four cocktails were formulated based on the host range of the phages to ensure coverage of as many bacterial strains as possible [Table 5]. We note that at this time, the phage genome sequences were not yet known. The incidence of American foulbrood is low and must be reported by law in New Zealand; the likelihood of a colony being infected with *P. larvae* in New Zealand is 0.0032^[55]^, so the chance of more than one strain infecting a single hive in New Zealand is extremely low (~0.00001024). Our phage cocktail design, therefore, focused on covering the breadth of strains that could infect a colony [Table 4].

**Table 5:** The phages contained within the four cocktails

**Cocktail one**	Callan	Logan	ApiWellbeing	Freya
**Cocktail two**	Dash	AJG77	UtuhinaGold_Zacery	NHScienceFair
**Cocktail three**	Callan	Ted	FutureBee	BarryFoster_Benicio
**Cocktail four**	Dash	LunBun	Carlos	Bloomfield

The initial set of cocktails, One to Four, were capable of lysing 93% of all *P. larvae* strains in our collection (28/30). Ultimately, each cocktail had a 70%-73% breadth of activity across our 30 bacterial isolates. The breadth of activity is calculated as the susceptibility of a pathogen to at least two phages in the cocktail^[56]^. Higher breadth is an indication of the cocktail's ability to mitigate phage resistance in the pathogen.

**Figure 6:**
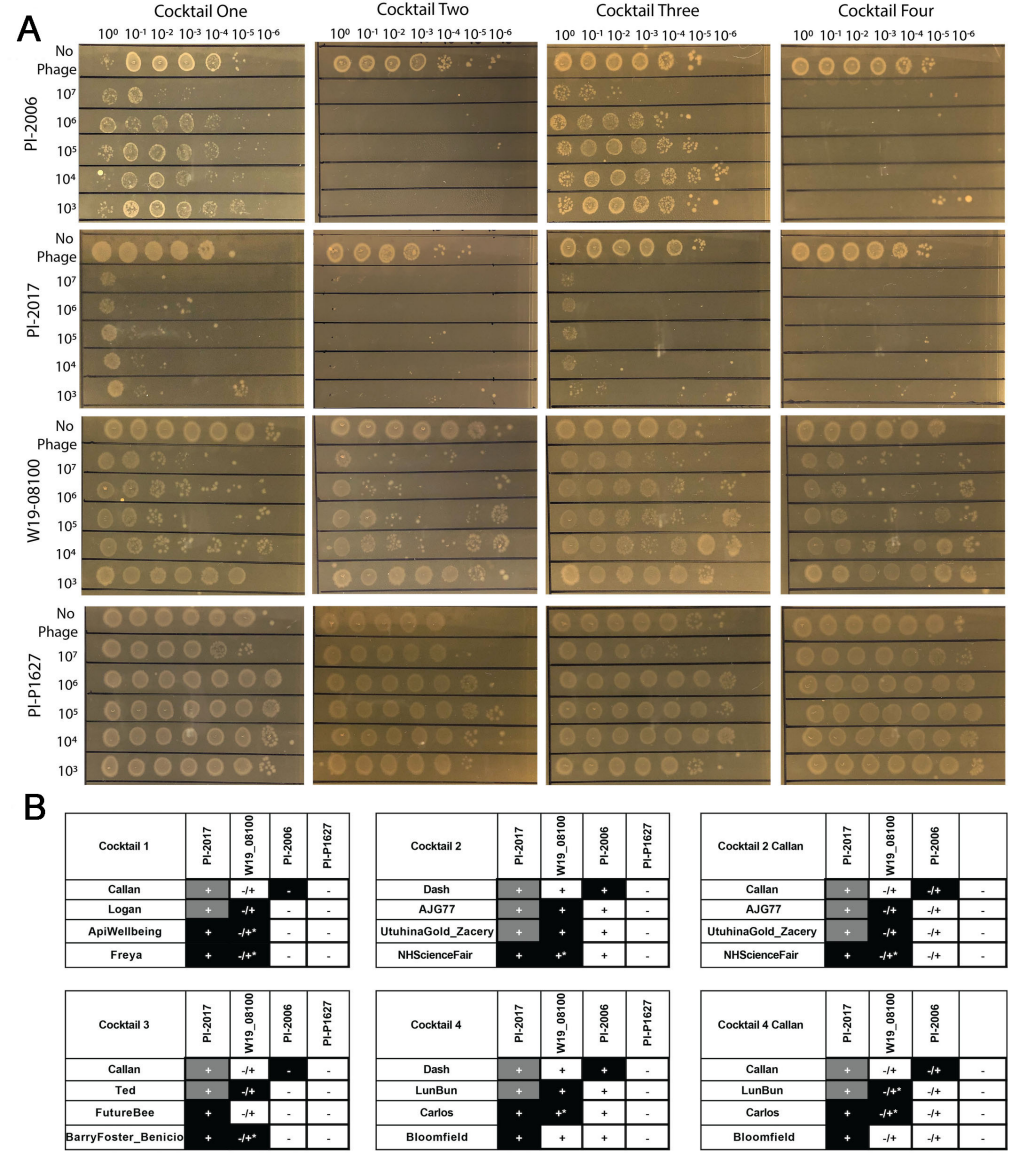
*In vitro* testing of phage cocktails. (A) Four cocktails were tested for their effectiveness against each of four bacterial strains: PFR-Pl-2017, PFR-Pl-2006, W19-08100, and Pl-P1627. Strain Pl-P1627 was known to be resistant to all phages in this study; (B) Tables to summarize the results of cocktail testing and the substitution of Phage Callan for Phage Dash in Cocktails 2 and 4. Table colours represent the host range observations. Black: plaques; grey: full clearings; white: no infection. The symbols within the table record the overall effectiveness of the cocktail that phage is in on that host strain: +: effective cocktail; -/+: partially effective cocktail; -: ineffective cocktail.

Once the four cocktails were decided upon, they were tested against four bacterial strains chosen to represent the types of *P. larvae* present in New Zealand; the bacterial strains selected were Pl-2017, Pl-2006, W19-08100, and Pl-P1627 [Figure 6A]. *P. larvae* Pl-P1627 was chosen as a negative control as it is not infected by the *P. larvae* phages in our collection to date.

All four cocktails showed good activity on strain Pl-2017, with cocktails Two and Four showing slightly better lysis potential. Cocktails Two and Four were very effective on strain Pl-2006, while cocktails One and Three only showed adequate lysis at the highest concentration of phages. On strain W19-08100, cocktails One, Two and Four all showed some lysis, while cocktail Three showed very little lysis potential on the susceptible *P. larvae* isolates. *P. larvae* Pl-P1627 was not infected by the cockails.

Ultimately, we sequenced the phage genomes and found that despite their excellent activity, phage cocktails Two and Four contained phage Dash, a phage with a large Plx1 toxin encoded in the genome. The host range of phage Dash was, however, very similar to that of phage Callan. These two Harrison cluster phages shared 91% of their genes and Callan does not contain the Plx1 toxin. We therefore tested the capacity of cocktails Two and Four with phage Callan on the same *P. larvae* strains [Supplementary Figure 3 and Figure 6B]. To our surprise, this seemingly small substitution did not retain the activity of the original cocktails Two and Four. Replacing phage Dash with the safer phage, phage Callan reduced the capacity of the cocktails to lyse these strains. This phenomenon warrants further investigation and suggests that phage-phage interactions are coming into play.

## DISCUSSION

In this study, we have set out to lay the groundwork for using phages as a prophylactic against the devastating pathogen *P. larvae* in the New Zealand apiculture industry. Our work began with the isolation and preliminary sequencing of a set of eight novel *P. larvae* strains from across New Zealand. These *P. larvae* bacterial strains were directly isolated from bee larvae or beehives with clinical signs of AFB. This allowed us to start the only collection of *P. larvae* bacterial strains available in New Zealand at the time. Previous New Zealand work had suggested that two separate ERIC genotypes (I and II) were present^[20]^. To take a cursory look, these *P. larvae* isolates were sequenced to between 157 to 219 contigs and were found to belong to either the 18 or 23 MLST. MLST 18 and MLST 23 are consistent with the ERIC I genotype^[36-38]^. To the best of our knowledge, there is no complete genome sequence data suggesting the presence of the ERIC II genotype in New Zealand today.

To better understand how these isolates might interact with phages, we screened these preliminary genomes for positive signs of phage defense mechanisms. We found each of our isolates contained CRISPR arrays. Each strain contained four to five CRISPR arrays with a total of 15 to 25 spacers. *P. larvae* strains isolated previously have been found to contain CRISPR arrays as phage defense mechanisms. *P. larvae* ERIC I strains ATCC 9545 and DSM 7030 contained four CRISPR arrays with 17 spacers^[57]^.

We also used PHASTER to evaluate the sequenced contigs for prophages, and we found each isolate had one or two intact prophages. In another study, *P. larvae* ERIC type I strains DSM 25719, MEX14, ATCC 9545, and DSM 7030 contained eight, three, five, and five intact prophages, respectively^[58]^. ATCC 9545 contained prophages similar to the prophages found in our *P. larvae* isolates.

Seven anti-phage systems were also discovered within our eight bacterial strains. These data suggested to us that these isolates are encountering an active population of phages in nature and are maintaining a suite of defense systems to counter infection when they meet. This is common as previous reports suggest that 50% of bacteria have CRISPR systems^[59]^ and other defense mechanisms such as restriction-modification systems are widely found within prokaryotes^[60]^.

The ApiWellbeing project, an initiative of the New Zealand Ministry for Primary Industries, generously gifted us with 22 additional *P. larvae* isolates from their own recent collection efforts, which brought our collection of hosts to 30^[38]^. The sequencing and annotation of this collection are underway and will provide a valuable asset in the future.

Since the discovery of the first *P. larvae* phage in 1953^[61]^, 69 *P. larvae-*specific phages have been found^[49,62-69]^. Due to the strict biosecurity laws in New Zealand, it is unlikely that non-native phages would be permitted in the apiculture industry here. We therefore sought to discover a suite of native New Zealand *P. larvae* phages to combat AFB.

Previous hunts for *P. larvae* phages have included samples from soil, bee debris, cosmetics, and bee wax^[62,65,69]^. A large-scale hunt across New Zealand was a daunting task for our small team; we, therefore, approached beekeepers from around New Zealand and received 430 samples of bee debris and soil from both the North and South Islands. These types of community science phage hunts have been used previously and there are ongoing community science projects to isolate new phages for *Pseudomonas aeruginosa*^[70]^. These samples were processed and led to the discovery of 26 independent phages. Unlike similar efforts overseas^[17,62]^ in which phages have been isolated from infected hives, the phages discovered herein were reported to have been isolated only from hive material or soil associated with healthy hives^[62]^.

The phage genomes were sequenced to completion, their genes were identified and annotated, and the genomes were made available publicly [Table 4]. Sequencing and annotation of phage genomes is particularly important for identifying gene functions that would make the phages unsuitable or unsafe for therapeutic use. In this study, we found that two phages, Dash and Lilo, contain a dangerous toxin that confers virulence to *P. larvae,* thereby ruling out these two phages for future therapy applications. Unfortunately, at the time the cocktail testing was carried out, these phages had not yet been sequenced, and the presence of this toxin was therefore unknown.

To determine whether our phage genomes were distinct, we used criteria previously described by Stamereilers *et al*.^[19]^. Phages are usually phenotypically identical if they have an ANI greater than 99.975%. We had several groups of phages that had ANI greater than this cut-off value. However, further analysis showed they all contained at least one amino acid difference, so in these instances, the phages were classed as phenotypically different. A more thorough analysis of these genomes and their relationships to the global *P. larvae* phages is in preparation.

Host range experiments revealed nine distinct infection patterns, which included a subset of six bacterial strains that were only able to be infected by three phages and two recalcitrant bacterial strains that were not infected by any of the phages discovered in New Zealand to date. Overall, there was a 93% host range coverage for our collection of *P. larvae* phages. In similar host-range experiments with *P. larvae* phages, Yost *et al.* found a 100% host-range coverage when testing 29 phages on 11 *P. larvae* bacterial strains^[17]^. In another experiment, Brady *et al*. tested 39 *P. larvae* specific phages on 59 bacterial strains and also found a 100% host range coverage^[18]^. Efforts are ongoing to find phages that can lyse the final resistant strains that are present in New Zealand. We do not currently know if phages found overseas have the ability to lyse the resistant *P. larvae* strains.

Four cocktails were formulated and tested against each of the four *P. larvae* isolates before the genomes were known. Cocktails One and Three both contained Phage Callan and Cocktails Two and Four both contained Phage Dash, as these were two of the phages that were able to infect six bacterial strains resistant to all other phages.

*In vitro* testing using these four cocktails varied, with two cocktails standing out as the most effective at killing three of the bacterial strains. One *P. larvae* strain, Pl-P1627, was completely resistant to all cocktails. This was to be expected as this strain was not infected by any of our individual phages. In this limited instance, we did not see any evidence of emergent infectivity above and beyond that of the individual phages present in the cocktail.

In our study, cocktails Two and Four were more effective than cocktails One and Three against *P. larvae* strains Pl-2006, Pl-2017 and W19-08100. In New Zealand, the likelihood of *P. larvae* infections is 0.0032^[55]^. Therefore, an ideal cocktail should be highly effective against each of the prevalent *P. larvae* strains, but it need not be effective against infection by multiple strains simultaneously at the level of a hive.

Interestingly, cocktails One and Four had a predicted Breadth_1_ of activity of 50% (2/4 phages were able to infect at least 2/4 bacterial strains), while cocktails Two and Three had a 75% Breadth_1_ of activity. This suggests that the quality of the phage cocktails tested here cannot be attributed to the Breadth_1_ of activity alone. The cause of the difference in outcomes of these phage cocktails remains to be investigated.

We observed host-dependent phage antagonism in our cocktails. When phage Callan was used in place of phage Dash on *P. larvae* W19-08100. This was surprising because phage Callan is not able to form plaques on this host. There are at least two possible explanations for this phenomenon. It may imply that the effect of the presence of Callan in the cocktail is the result of a direct host response preventing cocktail members from infection as a result of phage Callan DNA in the cytoplasm. Another possible mechanism is that the phage Callan lysate contains some effector which changes the susceptibility of this host to other phage cocktail members. Several studies have shown that phages within phage cocktails can have an antagonistic relationship. Forti *et al* found combining six phages into a cocktail to lyse *Pseudomonas aeruginosa* showed a lower host range than what had been predicted based on individual phage host ranges^[71]^. Another study testing different phage cocktails on *Escherichia coli* O157 showed that not all combinations of phages were as effective as others and phage antagonism was common in certain cocktails^[72]^. Our results suggest that there can be both host dependence on these antagonistic effects and that non-plaque forming phages can induce host resistance to infection. These preliminary results warrant further investigation.

These results, taken together with previous studies, show the importance of testing a variety of phage cocktails to find the most effective combination of phages, regardless of their individual host ranges. All *P. larvae* phages described to date have genomic features which suggest they are temperate^[49]^. Despite this observation, Brady *et al.* produced a phage cocktail containing three *P. larvae* phages and tested it on beehives at risk of AFB infection. This cocktail was able to protect the beehives for at least four months post-application^[18]^. These results indicate that even if a temperate lifecycle is genomically indicated, phages can work effectively as a protective measure against AFB. Our future work will, however, include extensive *in vitro* and field trials to confirm that there are no unintended negative consequences to the use of the phages. In addition, we plan to investigate adaptive laboratory evolution as a method for selecting phages that have lost the capacity for temperate life cycles.

This study shows promising results and forms the beginning of the work needed to find a solution to prophylactically protect honey bees in New Zealand from the destructive disease known as AFB. Further work will need to be undertaken to completely understand the characteristics of the phages, their persistence in the environment, cross-resistance of *P. larvae* strains to phages, and appropriate delivery mechanisms. In order to bring about a prophylactic solution for beekeepers, we will also need to undertake *in vitro* testing on honeybee larvae and large-scale field trials^[11]^. This work has been made possible by the collective efforts of the beekeepers of New Zealand, and we will continue to honour their contributions by pursuing this project further.

## DECLARATIONS

### Availability of data and materials

The authors confirm that the genomes used for this work are publicly available, and their accession numbers are listed in Tables 1 and 4.

### Copyright

© The Author(s) 2023.

## References

[1] Klein AM, Vaissière BE, Cane JH (2007). Importance of pollinators in changing landscapes for world crops. Proc Biol Sci.

[2] Newstrom-Lloyd LE http://www.mwpress.co.nz/__data/assets/pdf_file/0008/77057/2_11_Newstrom.pdf.

[3] https://www.mpi.govt.nz/dmsdocument/48793-2021-Apiculture-monitoring-report-data.

[4] Li G, Zhao H, Liu Z, Wang H, Xu B, Guo X (2018). The wisdom of honeybee defenses against environmental stresses. Front Microbiol.

[5] Genersch E (2010). American Foulbrood in honeybees and its causative agent, *Paenibacillus larvae*. J Invertebr Pathol.

[6] Genersch E (2008). *Paenibacillus larvae* and American Foulbrood - long since known and still surprising. J Verbr Lebensm.

[7] Alippi AM, Reynaldi FJ, López AC, De Giusti MR, Aguilar OM (2004). Molecular epidemiology of *Paenibacillus larvae larvae* and incidence of American Foulbrood in Argentinean honeys from Buenos Aires province. J Apic Res.

[8] Rauch S, Ashiralieva A, Hedtke K, Genersch E (2009). Negative correlation between individual-insect-level virulence and colony-level virulence of *Paenibacillus larvae*, the etiological agent of American Foulbrood of honeybees. Appl Environ Microbiol.

[9] Alippi AM, López AC, Aguilar OM (2022). Differentiation of *Paenibacillus larvae* subsp. *larvae*, the cause of American Foulbrood of honeybees, by using PCR and restriction fragment analysis of genes encoding 16S rRNA. Appl Environ Microbiol.

[10] Lester P https://play.google.com/books/reader?id=fY4bEAAAQBAJ&pg=GBS.PP1&hl=en.

[11] Kok DN, Hendrickson HL

[12] Goodwin M (2005). American Foulbrood control: the New Zealand approach. Bee World.

[13] https://afb.org.nz/wp-content/uploads/2018/07/BRIEFING-DOCUMENT-MPI-Government-01112017.pdf.

[14] https://afb.org.nz/wp-content/uploads/2018/10/Biosecurity-National-American-Foulbrood-Pest-Management-Plan-Order-1998.pdf.

[15] Mushegian AR (2020). Are there 10^31^ virus particles on earth, or more, or fewer?. J Bacteriol.

[16] Hendrix RW, Smith MC, Burns RN, Ford ME, Hatfull GF (1999). Evolutionary relationships among diverse bacteriophages and prophages: all the world’s a phage. Proc Natl Acad Sci USA..

[17] Yost DG, Tsourkas P, Amy PS (2016). Experimental bacteriophage treatment of honeybees (*Apis mellif era*) infected with *Paenibacillus larvae*, the causative agent of American Foulbrood Disease. Bacteriophage.

[18] Brady TS, Merrill BD, Hilton JA, Payne AM, Stephenson MB, Hope S (2017). Bacteriophages as an alternative to conventional antibiotic use for the prevention or treatment of *Paenibacillus larvae* in honeybee hives. J Invertebr Pathol.

[19] Stamereilers C, Fajardo CP, Walker JK (2018). Genomic analysis of 48 *Paenibacillus larvae *bacteriophages. Viruses.

[20] Graham SAM https://openaccess.wgtn.ac.nz/articles/thesis/American_foulbrood_and_its_causative_agent_Paenibacillus_larvae_in_New_Zealand_s_registered_hives_and_apiaries/17013008.

[21] Dingman DW, Stahly DP (1983). Medium promoting sporulation of *Bacillus larvae* and metabolism of medium components. Appl Environ Microbiol.

[22] de Graaf DC, Alippi AM, Antúnez K (2013). Standard methods for American Foulbrood research. J Apic Res.

[23] Dobbelaere W, de Graaf DC, Peeters JE (2001). Development of a fast and reliable diagnostic method for American Foulbrood disease (*Paenibacillus larvae* subsp. *larvae*) using a 16S rRNA gene based PCR. Apidologie.

[24] Bankevich A, Nurk S, Antipov D (2012). SPAdes: a new genome assembly algorithm and its applications to single-cell sequencing. J Comput Biol.

[25] Prjibelski A, Antipov D, Meleshko D, Lapidus A, Korobeynikov A (2020). Using SPAdes De Novo assembler. Curr Protoc Bioinformatics.

[26] Overbeek R, Olson R, Pusch GD (2014). The SEED and the Rapid Annotation of microbial genomes using Subsystems Technology (RAST). Nucleic Acids Res.

[27] Brettin T, Davis JJ, Disz T (2015). RASTtk: a modular and extensible implementation of the RAST algorithm for building custom annotation pipelines and annotating batches of genomes. Sci Rep.

[28] Aziz RK, Bartels D, Best AA (2008). The RAST Server: rapid annotations using subsystems technology. BMC Genomics.

[29] Seemann T (2014). Prokka: rapid prokaryotic genome annotation. Bioinformatics.

[30] Kok DN, Turnbull J, Takeuchi N, Tsourkas PK, Hendrickson HL (2023). *In Vitro* evolution to increase the titers of difficult bacteriophages: RAMP-UP protocol. Phage.

[31] Santos MA (1991). An improved method for the small scale preparation of bacteriophage DNA based on phage precipitation by zinc chloride. Nucleic Acids Res.

[32] Lazeroff M, Ryder G, Harris SL, Tsourkas PK (2021). Phage commander, an application for rapid gene identification in bacteriophage genomes using multiple programs. Phage.

[33] Pope WH, Jacobs-sera D

[34] Salisbury A, Tsourkas PK (2019). A method for improving the accuracy and efficiency of bacteriophage genome annotation. Int J Mol Sci.

[35] Jolley KA, Bray JE, Maiden MCJ (2018). Open-access bacterial population genomics: BIGSdb software, the PubMLST.org website and their applications. Wellcome Open Res.

[36] Morrissey BJ, Helgason T, Poppinga L, Fünfhaus A, Genersch E, Budge GE (2015). Biogeography of *Paenibacillus larvae*, the causative agent of American Foulbrood, using a new multilocus sequence typing scheme. Environ Microbiol.

[37] Papić B, Diricks M, Kušar D (2021). Analysis of the global population structure of *Paenibacillus larvae* and outbreak investigation of American Foulbrood using a stable wgMLST scheme. Front Vet Sci.

[38] Binney BM, Pragert H, Foxwell J (2023). Genomic analysis of the population structure of *Paenibacillus larvae* in New Zealand. Front Microbiol.

[39] Grissa I, Vergnaud G, Pourcel C (2007). CRISPRFinder: a web tool to identify clustered regularly interspaced short palindromic repeats. Nucleic Acids Res.

[40] Tesson F, Hervé A, Mordret E (2022). Systematic and quantitative view of the antiviral arsenal of prokaryotes. Nat Commun.

[41] Abby SS, Néron B, Ménager H, Touchon M, Rocha EP (2014). MacSyFinder: a program to mine genomes for molecular systems with an application to CRISPR-Cas systems. PLoS One.

[42] Oliveira PH, Touchon M, Rocha EP (2014). The interplay of restriction-modification systems with mobile genetic elements and their prokaryotic hosts. Nucleic Acids Res.

[43] Gao L, Altae-Tran H, Böhning F (2020). Diverse enzymatic activities mediate antiviral immunity in prokaryotes. Science.

[44] Bernheim A, Bikard D, Touchon M, Rocha EPC (2020). Atypical organizations and epistatic interactions of CRISPRs and cas clusters in genomes and their mobile genetic elements. Nucleic Acids Res.

[45] Doron S, Melamed S, Ofir G (2018). Systematic discovery of antiphage defense systems in the microbial pangenome. Science.

[46] Millman A, Melamed S, Leavitt A (2022). An expanded arsenal of immune systems that protect bacteria from phages. Cell Host Microbe.

[47] Arndt D, Grant JR, Marcu A (2016). PHASTER: a better, faster version of the PHAST phage search tool. Nucleic Acids Res.

[48] Zhou Y, Liang Y, Lynch KH, Dennis JJ, Wishart DS (2011). PHAST: a fast phage search tool. Nucleic Acids Res.

[49] Tsourkas PK, Yost DG, Krohn A (2015). Complete genome sequences of nine phages capable of infecting *Paenibacillus larvae*, the causative agent of American Foulbrood disease in honeybees. Genome Announc.

[50] Tsourkas PK (2020). *Paenibacillus larvae* bacteriophages: obscure past, promising future. Microb Genom.

[51] Ebeling J, Fünfhaus A, Genersch E (2021). The buzz about ADP-ribosylation toxins from *Paenibacillus larvae*, the causative agent of American Foulbrood in honey bees. Toxins.

[52] Fünfhaus A, Poppinga L, Genersch E (2013). Identification and characterization of two novel toxins expressed by the lethal honey bee pathogen *Paenibacillus larvae*, the causative agent of American Foulbrood. Environ Microbiol.

[53] Ackermann HW (2003). Bacteriophage observations and evolution. Res Microbiol.

[54] https://www.mpi.govt.nz/biosecurity/how-to-find-report-and-prevent-pests-and-diseases/bee-biosecurity/apiwellbeing/.

[55] King C (2020). American foulbrood. Surveillance.

[56] Abedon ST, Danis-Wlodarczyk KM, Wozniak DJ (2021). Phage cocktail development for bacteriophage therapy: toward improving spectrum of activity breadth and depth. Pharmaceuticals.

[57] Stamereilers C, Wong S, Tsourkas PK (2021). Characterization of CRISPR spacer and protospacer sequences in *Paenibacillus larvae* and its bacteriophages. Viruses.

[58] Ribeiro HG, Nilsson A, Melo LDR, Oliveira A (2022). Analysis of intact prophages in genomes of *Paenibacillus larvae*: an important pathogen for bees. Front Microbiol.

[59] Hille F, Richter H, Wong SP, Bratovič M, Ressel S, Charpentier E (2018). The biology of CRISPR-Cas: backward and forward. Cell.

[60] Loenen WA, Raleigh EA (2014). The other face of restriction: modification-dependent enzymes. Nucleic Acids Res.

[61] Gochnauer T A (1970). Some properties of a bacteriophage from *Bacillus larvae*. J Invertebr Pathol.

[62] Merrill BD, Fajardo CP, Hilton JA (2018). Complete genome sequences of 18 *Paenibacillus larvae *phages from the western United States. Microbiol Resour Announc.

[63] Jończyk-Matysiak E, Owczarek B, Popiela E (2021). Isolation and characterization of phages active against *Paenibacillus larvae *causing American Foulbrood in honeybees in Poland. Viruses.

[64] Carson S, Bruff E, DeFoor W (2015). Genome sequences of six *Paenibacillus larvae* siphoviridae phages. Genome Announc.

[65] Walker JK, Merrill BD, Berg JA (2018). Complete genome sequences of *Paenibacillus larvae *phages BN12, Dragolir, Kiel007, Leyra, Likha, Pagassa, PBL1c, and Tadhana. Genome Announc.

[66] Beims H, Wittmann J, Bunk B (2015). *Paenibacillus larvae*-directed bacteriophage HB10c2 and its application in American Foulbrood-affected honey bee larvae. Appl Environ Microbiol.

[67] Oliveira A, Melo LD, Kropinski AM, Azeredo J (2013). Complete genome sequence of the broad-host-range *Paenibacillus larvae *phage phiIBB_Pl23. Genome Announc.

[68] Ribeiro HG, Melo LDR, Oliveira H (2019). Characterization of a new podovirus infecting *Paenibacillus larvae*. Sci Rep.

[69] Yost DG, Chang C, LeBlanc L (2018). Complete genome sequences of *Paenibacillus larvae* phages halcyone, heath, scottie, and unity from Las Vegas, Nevada. Microbiol Resour Announc.

[70] https://www.citizenphage.com.

[71] Forti F, Roach DR, Cafora M (2018). Design of a broad-range bacteriophage cocktail that reduces *Pseudomonas aeruginosa* biofilms and treats acute infections in two animal models. Antimicrob Agents Chemother.

[72] Niu YD, Liu H, Du H (2021). Efficacy of individual bacteriophages does not predict efficacy of bacteriophage cocktails for control of *Escherichia coli* O157. Front Microbiol.

