# Supplementary material for: Paenibacillus larvae and their phages; a community science approach to discovery and initial testing of prophylactic phage cocktails against American Foulbrood in New Zealand
Source: Microbiome Res Rep. 2023 Aug 1;2(4):30. doi: 10.20517/mrr.2023.16 (PMC10688787; doi:10.20517/mrr.2023.16)

Supplementary Materials

Supplementary Figure 1. An infographic was distributed to beekeepers to inform them of the research being undertaken and to request their help in sourcing samples.

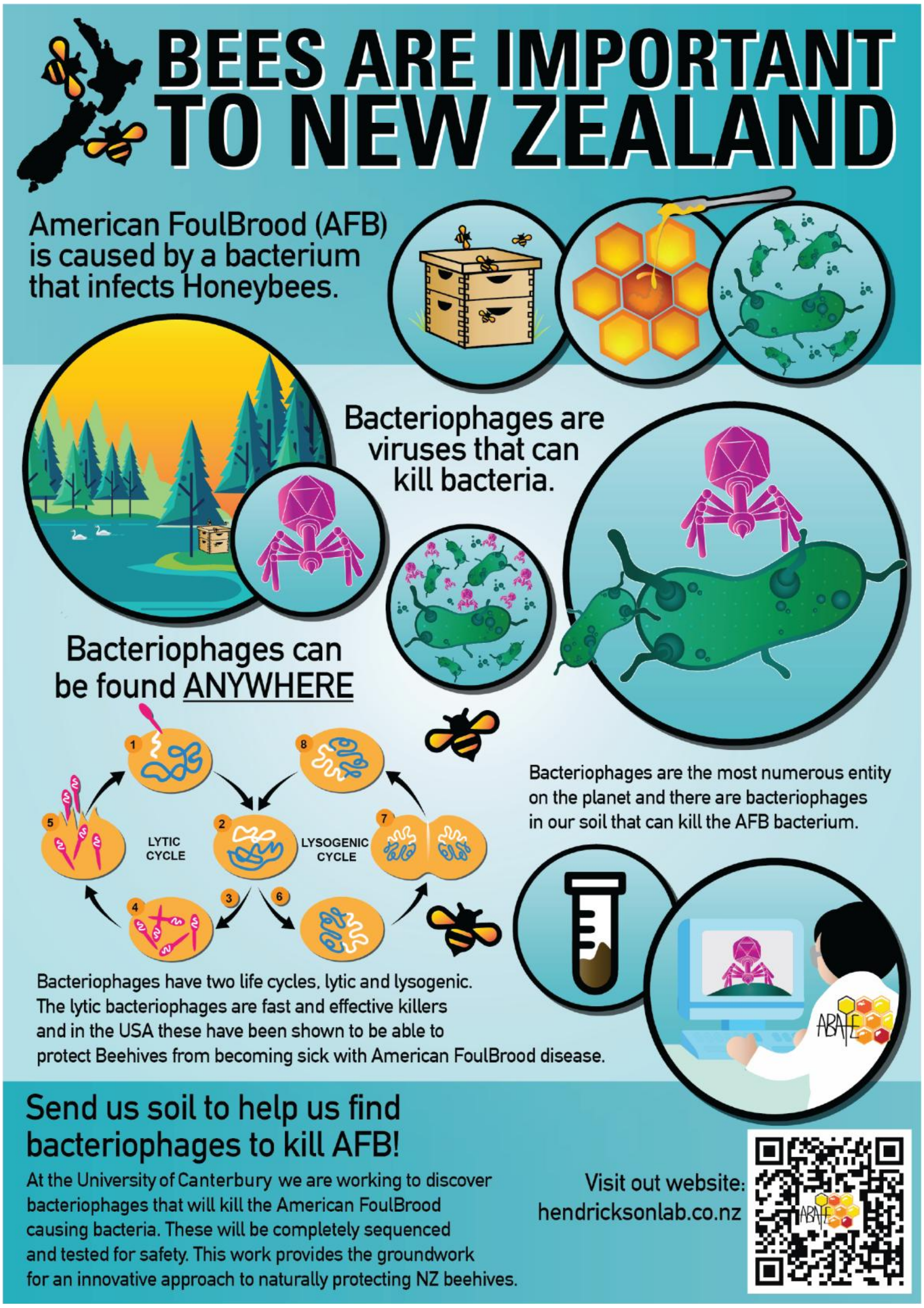

Supplementary Figure 2. Host range of *P. larvae* phages on *P. larvae* bacterial isolates from New Zealand. Black boxes indicate full spot clearing, grey boxes indicate individual plaques were observed, and white boxes indicate no cell lysis has occurred.

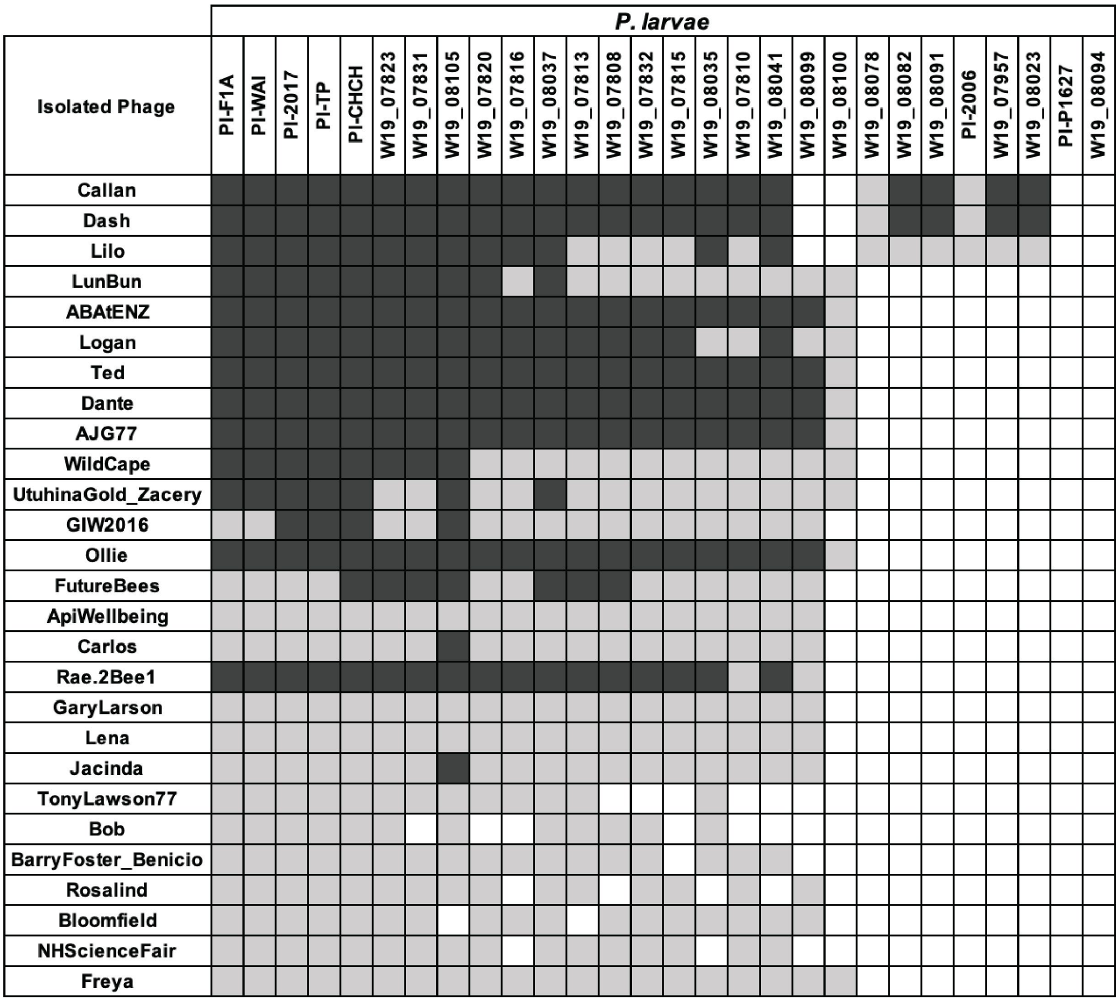

Supplementary Figure 3. Cocktail two and cocktail tested for their effectiveness against each of four bacterial strains; PFR-Pl-2017, PFR-Pl-2006, W19-08100 and Pl-P1627 with the substitution of phage Callan for phage Dash.

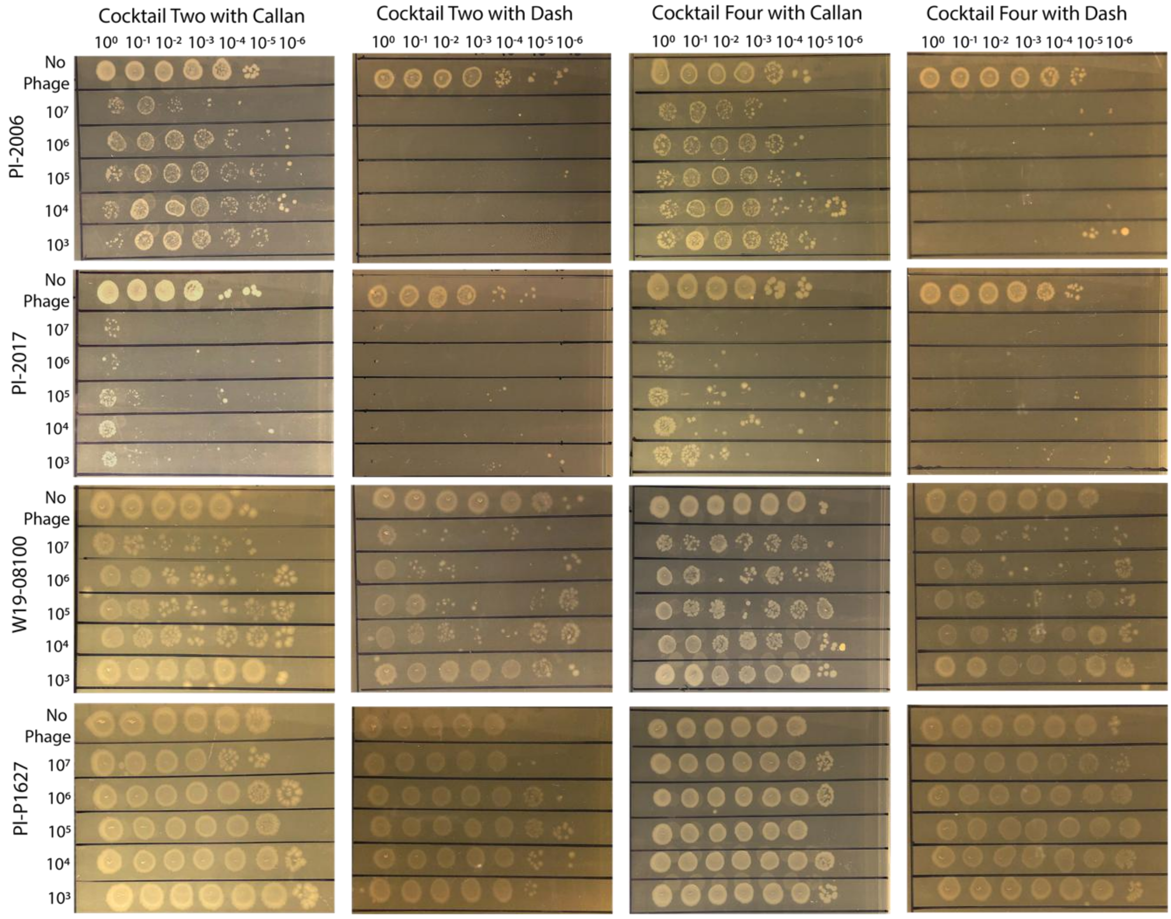

Supplement: Supplementary file 1 [file mrr-2-4-30-SupplementaryMaterials.pdf]
